# Supplementary material for: Functional Interactomes of Genes Showing Association with Type-2 Diabetes and Its Intermediate Phenotypic Traits Point towards Adipo-Centric Mechanisms in Its Pathophysiology
Source: Biomolecules. 2020 Apr 13;10(4):601. doi: 10.3390/biom10040601 (PMC7226597; doi:10.3390/biom10040601)
Supplement: Supplementary file 1 [file biomolecules-10-00601-s001.zip › Supplementary Table 4.docx]

| **Supplementary table 4: Enriched pathway implicated by DEGs-HOMA B genes in various insulin responsive tissues** | | | | | | |
| --- | --- | --- | --- | --- | --- | --- |
| **Adipose - HOMA-B** | | | | | | |
| **Gene Set** | **Description** | **Size** | **Expect** | **Ratio** | **P Value** | **FDR** |
| hsa04068 | FoxO signaling pathway | 132 | 0.94402 | 10.593 | 2.2868E-08 | 7.5E-06 |
| hsa04931 | Insulin resistance | 107 | 0.76523 | 11.761 | 5.008E-08 | 8.2E-06 |
| hsa05206 | MicroRNAs in cancer | 150 | 1.0728 | 8.3896 | 9.1903E-07 | 1E-04 |
| hsa04152 | AMPK signaling pathway | 120 | 0.8582 | 9.3218 | 1.7863E-06 | 0.00015 |
| hsa05213 | Endometrial cancer | 58 | 0.4148 | 14.465 | 3.0317E-06 | 0.0002 |
| hsa04910 | Insulin signaling pathway | 137 | 0.97978 | 8.1651 | 4.8446E-06 | 0.00026 |
| hsa04920 | Adipocytokine signaling pathway | 69 | 0.49347 | 12.159 | 8.4574E-06 | 0.00031 |
| hsa05205 | Proteoglycans in cancer | 198 | 1.416 | 6.3558 | 9.1627E-06 | 0.00031 |
| hsa04917 | Prolactin signaling pathway | 70 | 0.50062 | 11.985 | 9.2001E-06 | 0.00031 |
| hsa05219 | Bladder cancer | 41 | 0.29322 | 17.052 | 9.4814E-06 | 0.00031 |
| **Pancreas- HOMA-B** | | | | | | |
| **Gene Set** | **Description** | **Size** | **Expect** | **Ratio** | **P Value** | **FDR** |
| hsa05206 | MicroRNAs in cancer | 150 | 1.4028 | 9.9798 | 6.5018E-11 | 2.1E-08 |
| hsa04068 | FoxO signaling pathway | 132 | 1.2345 | 10.531 | 1.7213E-10 | 2.7E-08 |
| hsa05205 | Proteoglycans in cancer | 198 | 1.8517 | 8.1005 | 2.4662E-10 | 2.7E-08 |
| hsa05200 | Pathways in cancer | 524 | 4.9006 | 4.4893 | 8.4011E-10 | 6.8E-08 |
| hsa05215 | Prostate cancer | 97 | 0.90717 | 12.126 | 1.0953E-09 | 7.1E-08 |
| hsa01521 | EGFR tyrosine kinase inhibitor resistance | 79 | 0.73883 | 13.535 | 2.2373E-09 | 1.2E-07 |
| hsa05210 | Colorectal cancer | 86 | 0.80429 | 12.433 | 5.2242E-09 | 2.4E-07 |
| hsa04071 | Sphingolipid signaling pathway | 118 | 1.1036 | 9.9677 | 9.0432E-09 | 3.7E-07 |
| hsa04151 | PI3K-Akt signaling pathway | 354 | 3.3107 | 5.1349 | 1.5281E-08 | 5.5E-07 |
| hsa04218 | Cellular senescence | 160 | 1.4964 | 8.0195 | 2.1416E-08 | 7E-07 |
| **Skeletal- HOMA-B** | | | | | | |
| **Gene Set** | **Description** | **Size** | **Expect** | **Ratio** | **P Value** | **FDR** |
| hsa04510 | Focal adhesion | 199 | 1.56 | 7.0511 | 3.0033E-07 | 4.6E-05 |
| hsa04152 | AMPK signaling pathway | 120 | 0.94072 | 9.5671 | 3.1075E-07 | 4.6E-05 |
| hsa04015 | Rap1 signaling pathway | 206 | 1.6149 | 6.8115 | 4.2559E-07 | 4.6E-05 |
| hsa05215 | Prostate cancer | 97 | 0.76042 | 10.521 | 7.2365E-07 | 5.9E-05 |
| hsa05418 | Fluid shear stress and atherosclerosis | 138 | 1.0818 | 8.3192 | 1.0221E-06 | 6.7E-05 |
| hsa04931 | Insulin resistance | 107 | 0.83881 | 9.5373 | 1.5365E-06 | 8.3E-05 |
| hsa01521 | EGFR tyrosine kinase inhibitor resistance | 79 | 0.61931 | 11.303 | 2.333E-06 | 0.00011 |
| hsa04068 | FoxO signaling pathway | 132 | 1.0348 | 7.731 | 7.4628E-06 | 0.0003 |
| hsa04910 | Insulin signaling pathway | 137 | 1.074 | 7.4488 | 9.8274E-06 | 0.00036 |
| hsa04066 | HIF-1 signaling pathway | 100 | 0.78394 | 8.9293 | 1.1346E-05 | 0.00037 |
| **Pancreas-skeletal-Adipose** | | | | | | |
| **Gene Set** | **Description** | **Size** | **Expect** | **Ratio** | **P Value** | **FDR** |
| hsa05418 | Fluid shear stress and atherosclerosis | 138 | 0.93 | 8.6022 | 3.2157E-06 | 0.00105 |
| hsa04020 | Calcium signaling pathway | 183 | 1.2333 | 5.676 | 0.00020041 | 0.03267 |
| hsa04979 | Cholesterol metabolism | 50 | 0.33696 | 11.871 | 0.00033387 | 0.03628 |
| hsa05120 | Epithelial cell signaling in Helicobacter pylori infection | 68 | 0.45826 | 8.7287 | 0.0010802 | 0.07852 |
| hsa04917 | Prolactin signaling pathway | 70 | 0.47174 | 8.4793 | 0.0012042 | 0.07852 |
